# Gender differences of foot characteristics in older Japanese adults using a 3D foot scanner

**DOI:** 10.1186/s13047-015-0087-4

**Published:** 2015-07-16

**Authors:** Mahshid Saghazadeh, Naruki Kitano, Tomohiro Okura

**Affiliations:** Faculty of Health and Sport Sciences, Graduate School of Comprehensive Human Sciences, University of Tsukuba, 1-1-1 Tennodai, Tsukuba, Ibaraki 305-8574 Japan

**Keywords:** Anthropometry, Elderly, Footwear, Shoe design, Three-dimensional shape

## Abstract

**Background:**

Knowledge of gender differences in foot shape assists shoe manufactures with designing appropriate shoes for men and women. Although gender differences in foot shapes are relatively known among young men and women, less is known about how the older men and women’s feet differ in shape. A recent development in foot shape assessment is the use of 3D foot scanners. To our knowledge this technology has yet to be used to examine gender differences in foot shape of Japanese older adults.

**Methods:**

This cross-sectional study included 151 older men (74.5 ± 5.6 years) and 140 older women (73.9 ± 5.1 years) recruited in Kasama City, Japan. Foot variables were measured in sitting and standing positions using Dream GP Incorporated’s 3D foot scanner, Footstep PRO (Osaka, Japan). Scores were analyzed as both raw and normalized to truncated foot length using independent samples *t*-test and analysis of covariance, respectively.

**Results:**

In men, the measurement values for navicular height, first and fifth toe and instep heights, ball and heel width, ball girth, arch height index (just standing), arch rigidity index and instep girth were significantly greater than the women’s, whereas the first toe angle, in both sitting and standing positions was significantly smaller. However, after normalizing, the differences in ball width, heel width, height of first and fifth toes in both sitting and standing and ball girth in sitting position were nonsignificant. According to Cohen’s d, among all the foot variables, the following had large effect sizes in both sitting and standing positions: truncated foot length, instep, navicular height, foot length, ball girth, ball width, heel width and instep girth.

**Conclusion:**

This study provides evidence of anthropometric foot variations between older men and women. These differences need to be considered when manufacturing shoes for older adults.

## Background

Knowledge of gender differences in foot shape and anatomy helps shoe manufacturers design appropriate shoes for men and women [1]. For instance, knowledge of the location of the metatarsophalangeal joint can help when deciding which areas of the shoe should be flexible or stiff [2]. Although information on foot shape differences between young men and women is available, there is much less information on how older men and women’s feet differ. One study compared the length and width of the feet of 668 older adults and concluded that more than two thirds of the feet were broader than the shoes available in their sizes [3]. Most shoe manufacturers utilize young adults’ feet data for their shoe designs [4]. Moreover, women’s shoes have traditionally been designed as a smaller version of men’s shoes with all dimensions proportionally scaled according to foot length. However, if women’s feet differ in shape from men’s feet, this is an inappropriate model for a woman’s shoe and could lead to improper shoe fit in women [5].

According to the literature, younger people tend to have smaller foot circumferences compared to older people [6]. Although, the elderly are reported to have flatter, longer, and wider feet than young adults [5, 7], we found only two studies, in Brazil and Australia, relating to such gender-related differences in older adults [4, 8]. However, in the Brazil study, all the foot measurements were obtained by caliper and goniometer [4]. In that study, women had significantly greater ball width and toe perimeters, while the heel width was significantly smaller relative to the height of the dorsal foot after normalizing the data to foot length. In addition, the first and fifth metatarsophalangeal angles were smaller in the men [4].

In the study conducted in Australia, the researchers measured foot anthropometrics using a calibrated 3D foot scanner. In that study, men had significantly larger measurements than the women for all dimensions with the exception of first toe angle. Men had significantly higher normalized first and fifth toe heights and a larger fifth toe angle, whereas women had a significantly longer normalized medial ball length and larger first toe angle [8]. In addition to gender differences, ethnic origin can also influence foot shape [9]. Therefore, studying older adults’ foot characteristic in each nation is indispensable [10].

The opportunity for podiatric research has improved in recent years with the new laser scanner technologies available for various applications [11]. This new technology with adequate speed of data capture provides the opportunity to quickly measure the three-dimensional shape of the foot in large populations. This can improve our ability to analyze gender differences more accurately. To the best of our knowledge, this technology has yet to be used for examining gender differences in foot shape of Japanese older adults.

Unfortunately, although inappropriate footwear is a known risk factor for falls of the elderly [12], little is known about what actually constitutes safe footwear for this age group [13]. Knowing the characteristics of older adults’ feet, including gender and ethnic differences, could improve footwear design and may reduce the risk of falls in the elderly. The main objective of this study was to determine gender differences in foot characteristics in a large community sample of older Japanese adults using the recently launched technology of 3D foot scanning. We could find no previous studies on this topic in Japan; this is the first such study.

## Methods

### Participants

We conducted this cross-sectional study in August 2012 in Kasama City (population 79,266, proportion of older adults 24.0 %), a rural region in Ibaraki prefecture, Japan.

A total of 349 older adults participated in this study conducted in the Kasama City health center. Of these participants, we excluded 58 due to incomplete data, their reliance on walking sticks during the measurement or among women because of refusing to remove their pantyhose preventing us from collecting foot characteristic. There were 151 men (74.5 ± 5.6 years) and 140 women (73.9 ± 5.1 years) participants for final data analysis. Medical histories and demographics variables are shown in Table 1. All participants provided a signed, informed consent. This study was approved by the Ethical Committee of University of Tsukuba.

**Table 1:** Participant characteristics

Variables	Men = 151		Women = 140	
	Mean or %	SD or Number	Mean or %	SD or Number
Age (years)	74.54	5.58	73.89	5.14
Height (cm)	162.62	5.73	148.94	5.67
Weight (kg)	61.28	8.25	51.38	7.4
BMI (kg/m^2^)	23.15	2.15	23.14	2.97
Diabetes	14.6 %	*N* = 22	12.1 %	*N* = 17
Osteoporosis	0.7 %	*N* = 1	16.4 %	*N* = 23

### Measurements

#### Foot characteristics

**Fig. 1:**
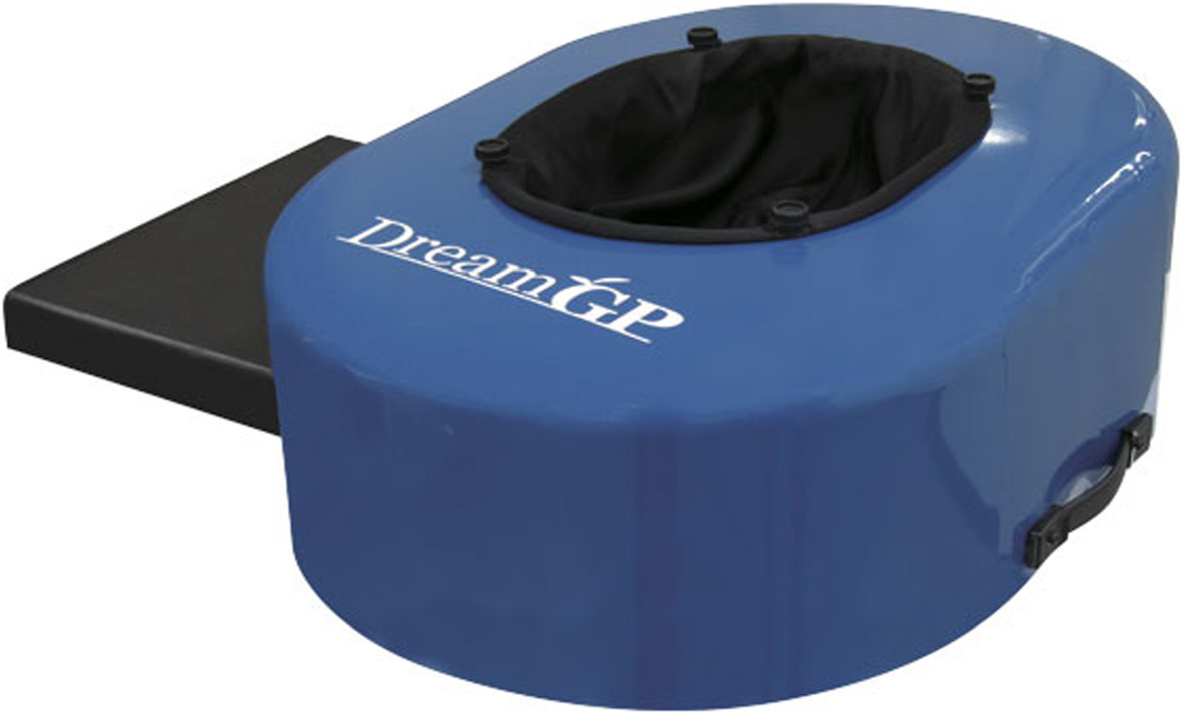
3D foot scanner, Footstep PRO by Dream GP Company, Osaka, Japan

**Fig. 2:**
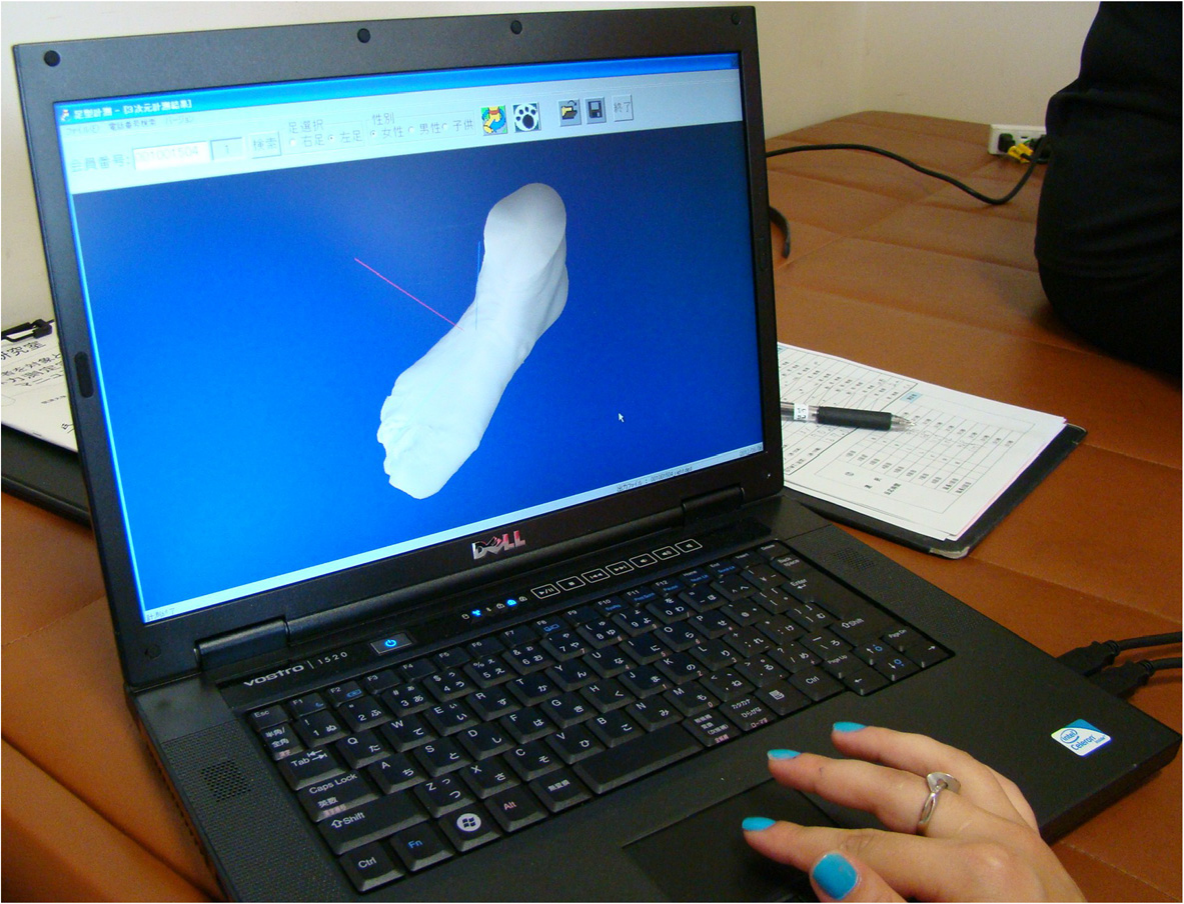
An example of 3D image by Footstep PRO

We measured foot characteristics using the recently launched 3D foot scanner, Footstep PRO by Dream GP Company, Osaka, Japan (Fig. 1). Modern 3D surface scanning systems can obtain accurate and repeatable digital representations of the foot shape and have been used successfully in medical, ergonomic and footwear development applications [14]. An example of 3D image by Footstep PRO is shown in Fig. 2.

Subjects individually sat with bare feet on the end of a table so their lower legs were non-weight bearing and their ankles were slightly plantar-flexed [15]. They placed their right feet onto the factory-delineated center of the scanner as the measurer assured proper positioning. To prevent ankle dorsiflexion, the subjects were instructed not to forcibly push the platform of the 3D machine [15]. Prior to starting the machine, light blocking material attached to the rim of the scanner was secured to subjects’ lower legs.

When the scanner is started, a laser rotates on the rail around the foot measuring about 30,000 positions, including instep, heel, sole and toe, which allows the software to reproduce exactly the shape of the foot. Each measurement is completed in about 13 s.

After completing measurement in a sitting position, participants stood up without changing their foot position inside the machine, set their left foot on an adjacent wooden platform next to and level with the platform inside the scanner and placed equal weight on each foot. This placed 50 % of their body weight on the foot being assessed. The measurer checked the foot positioning in the scanner prior to starting the machine. Participants were also encouraged to use the handrail placed in front of them for balance, to relax their feet and to ensure equal loading on each extremity. The handrail was placed at a level which they could easily reach without needing to raise or lower their arms too much. The participants looked straight ahead and stood as still as possible.

**Fig. 3:**
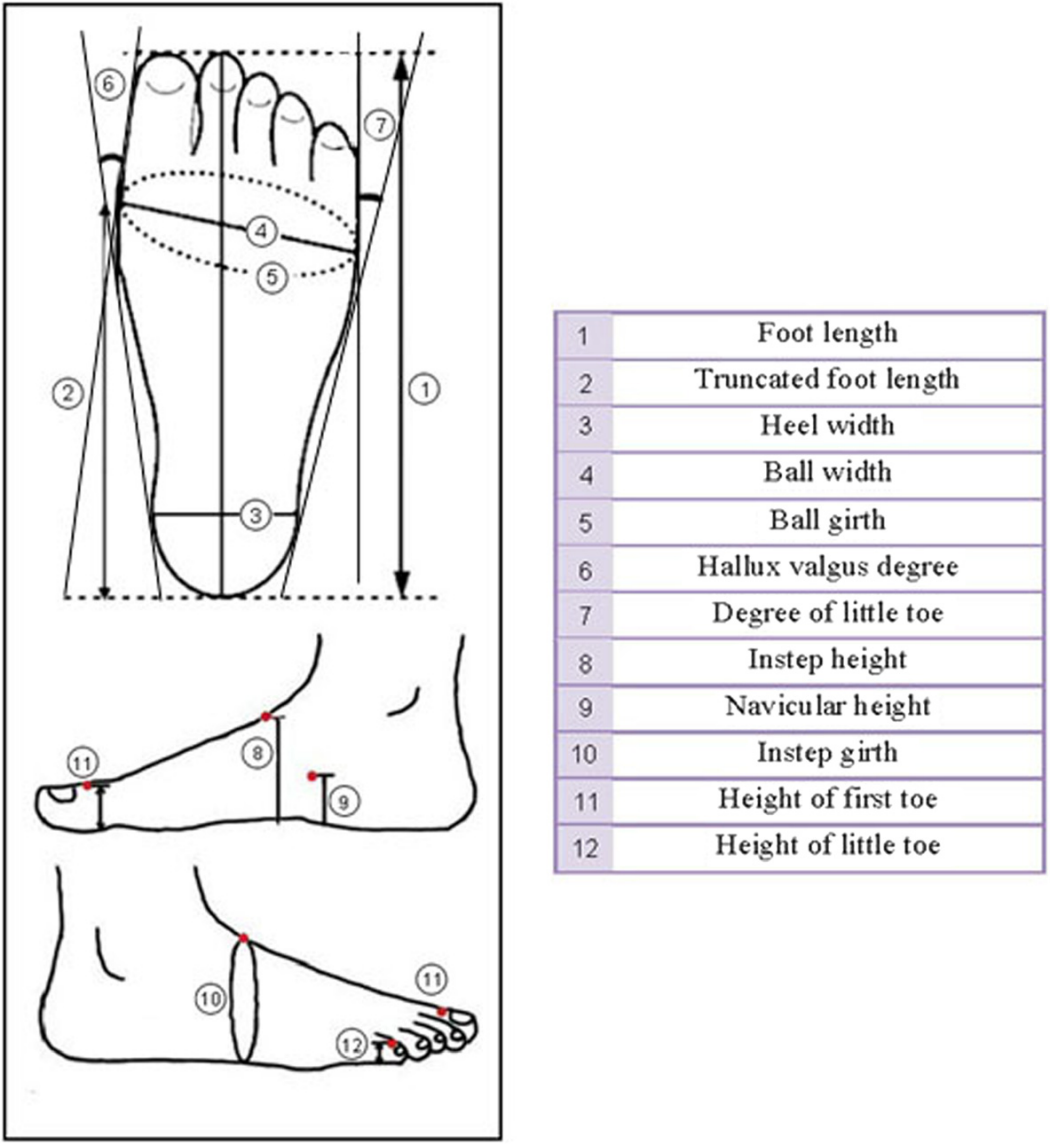
Foot characteristics

Once we obtained readings for the right foot in both sitting and standing positions, we repeated the measurements for the left foot. We collected 4 measurements on each person, right and left leg in both sitting and standing positions and then sanitized the instruments with 70 % alcohol prior to measuring the next person. Foot characteristics are shown in Fig. 3.

In this study, we used the following two methods for calculating arch height:

### Navicular height (NH) and navicular drop (ND)

We measured navicular heights as described in the literature [16]. The subject sat on a chair with bare feet. The most prominent portion of the navicular tuberosities on both feet were palpated and marked with a small, round, black sticky point while the participants maintained a relaxed sitting position. The 3D scanner software located these black points as the point of the navicular.

One investigator (MS) performed all markings of the navicular tuberosity. This investigator was a licensed athletic trainer with 2 years’ experience in foot and posture assessment at the time of testing. In this study, navicular height was defined as the linear distance (mm) from the most medial prominence of the navicular tuberosity to the supporting surface while sitting and while standing with 50 % body weight on each foot [17].

We defined navicular drop or foot mobility as the amount of vertical navicular excursion (mm) between the positions of the subtalar joint while neutral in sitting position and relaxed in bilateral standing (navicular drop) [18].

### Arch height index (AHI) and arch rigidity index (ARI)

Since skin markers over the navicular tuberosity have been shown to not track the actual position of the bone with complete accuracy [19], AHI was also used.

In this study, AHI was defined as the linear distance (mm) from the instep as defined by the foot scanning machine, to the supporting surface while sitting and while standing with 50 % weight bearing on each foot, divided by the truncated foot length [20].

Arch rigidity index (ARI) is defined as the ratio of standing AHI divided by seated AHI (AHI stand/AHI sit). Values nearer to 1 indicate a stiffer (less flexible) foot [21].

All the foot data were collected automatically by the Footstep PRO. However, some of the data such as foot length, navicular height, ball width and angle of first and fifth toes were adjustable by defining their points in the software. For instance, we defined foot length in this study as a linear distance from the most prominent point of the calcaneal tuberosity to the tip of the longest toe. The first or second toe was chosen as the longest after viewing the 3D foot shape with the software.

### Statistical analysis

Scores were analyzed as both raw and normalized to truncated foot length using independent samples *t*-test and analysis of covariance, respectively. Williams and McClay [22] indicated that using the truncated foot length, the perpendicular distance from the first metatarsophalangeal joint to the most posterior aspect of the heel, reduces the impact that toe deformities, such as claw toes and hallux valgus, may have on heel to longest toe foot length. Therefore, truncated foot length was used to normalize the data.

A P value of less than 0.001 was considered statistically significant. For each subject, we averaged the right and left foot measurements for the analyses. Cohen’s d is interpreted as a very small effect at less than 0.2, as a small effect between 0.2 to 0.5, as a moderate effect between 0.5 to 0.8, and as a large effect greater than 0.8. Statistical analyses were performed using SPSS version 18.0.

## Results

In men, the measurement values for navicular height, first and fifth toe and instep heights, ball and heel width, ball girth, AHI (just standing), ARI and instep girth were significantly greater than the women’s, whereas the first toe angle, in both sitting and standing positions was significantly smaller. However, after normalizing, the differences in ball width, heel width, height of first and fifth toes in both sitting and standing and ball girth in sitting position were nonsignificant. According to Cohen’s d, among all the foot variables, the following had large effect sizes in both sitting and standing positions: truncated foot length, instep, navicular height, foot length, ball girth, ball width, heel width and instep girth (Table 2).

**Table 2:** Independent sample *t*-test, ANCOVA (adjusted to truncated foot length) & Cohen’s d effect size

Foot characteristics	Men (*N* = 151) Mean ± SD	Women (*N* = 140) Mean ± SD	*t* test *P* value	ANCOVA *P* value	Effect size (ES)	Guide of ES
Sitting truncated foot length (mm)	179.81 ± 7.46	165.25 ± 6.92	*P* < 0.001	--	2.03	Large
Sitting instep (mm)	66.18 ± 4.30	59.65 ± 3.87	*P* < 0.001	*P* < 0.001	1.60	Large
Sitting NH (mm)	48.23 ± 6.21	42.15 ± 5.14	*P* < 0.001	*P* < 0.001	1.07	Large
Sitting AHI	0.37 ± 0.03	0.36 ± 0.03	.021	--	0.27	Small
Sitting foot length (mm)	243.19 ± 9.07	224.69 ± 8.86	*P* < 0.001	--	2.07	Large
Sitting ball girth (mm)	244.28 ± 11.65	227.95 ± 10.66	*P* < 0.001	.002	1.47	Large
Sitting ball width (mm)	98.93 ± 5.08	92.55 ± 5.24	*P* < 0.001	.490	1.24	Large
Sitting heel width (mm)	64.51 ± 4.22	59.93 ± 3.22	*P* < 0.001	.291	1.22	Large
Sitting first toe angle (degree)	10.42 ± 4.64	13.64 ± 6.92	*P* < 0.001	*P* < 0.001	0.55	Moderate
Sitting little toe angle (degree)	13.80 ± 4.00	13.35 ± 4.87	.393	.782	0.10	Very small
Sitting first toe height (mm)	18.03 ± 2.31	16.45 ± 2.16	*P* < 0.001	.057	0.71	Moderate
Sitting little toe height (mm)	12.88 ± 2.25	11.94 ± 1.85	*P* < 0.001	.564	0.46	Small
Sitting instep girth (mm)	248.93 ± 11.54	227.70 ± 10.85	*P* < 0.001	*P* < 0.001	1.90	Large
Standing foot length (mm)	246.20 ± 9.05	227.57 ± 9.13	*P* < 0.001	--	2.06	Large
Standing truncated foot length (mm)	182.16 ± 7.81	168.45 ± 7.01	*P* < 0.001	--	1.85	Large
Standing instep (mm)	61.68 ± 4.51	55.02 ± 3.88	*P* < 0.001	*P* < 0.001	1.58	Large
Standing NH (mm)	41.77 ± 6.32	35.76 ± 5.32	*P* < 0.001	*P* < 0.001	1.03	Large
Standing ball girth (mm)	247.09 ± 11.23	230.55 ± 11.41	*P* < 0.001	*P* < 0.001	1.47	Large
Standing ball width (mm)	101.53 ± 4.85	95.39 ± 5.74	*P* < 0.001	.251	1.16	Large
Standing heel width (mm)	65.89 ± 4.05	60.64 ± 4.67	*P* < 0.001	.006	1.21	Large
Standing first toe angle (degree)	10.73 ± 4.95	14.90 ± 7.60	*P* < 0.001	*P* < 0.001	0.63	Moderate
Standing little toe angle (degree)	13.84 ± 4.11	13.62 ± 5.00	.687	.725	0.05	Very Small
Standing first toe height (mm)	17.39 ± 1.91	16.02 ± 2.29	*P* < 0.001	.465	0.65	Moderate
Standing little toe height (mm)	12.76 ± 2.05	11.74 ± 1.87	*P* < 0.001	.084	0.52	Moderate
Standing instep girth (mm)	249.20 ± 11.05	228.30 ± 10.94	*P* < 0.001	*P* < 0.001	1.91	Large
Standing AHI	0.34 ± 0.03	0.33 ± 0.03	*P* < 0.001	--	0.46	Small
Arch drop (instep difference) (mm)	4.51 ± 1.70	4.65 ± 1.62	.464	.008	0.09	Very small
ND (mm)	6.46 ± 2.53	6.39 ± 2.53	.814	.610	0.03	Very small
ARI	0.92 ± 0.03	0.91 ± 0.03	*P* < 0.001	--	0.43	Small

## Discussion

This study demonstrates important anatomical differences of the foot between genders. Women have narrower feet in the heel and forefoot, and their instep, first and fifth toes and navicular height are also lower than men’s. Women also showed a greater first toe angle and lower ARI and AHI (just standing) compared to men. However, some of these differences were nonsignificant after normalizing to truncated foot length suggesting that the original findings were simply due to the fact that male feet tend to be larger than female feet. Furthermore, according to Cohen’s d, some differences were very small, and as a practical manner, the usefulness for shoe manufacturers to incorporate those differences is questionable. According to Wunderlich et al., these small differences may not even be perceptible when incorporated into footwear [10]. In our study, the first toe angle was significantly greater in women. The presence of hallux valgus can explain the larger values found among the women, because it occurs more frequently in women [23–25].

Our results were different in some respects to the gender difference studies in Brazil and Australia. In Brazil, unlike the results of our study, the width and perimeter of the toes and the width of the heel in the women were significantly greater than the men’s measurements. However, similar to our results, women had a significantly lower instep than men after normalizing the data to foot length, and the first and fifth metatarsophalangeal angles were smaller in the men [4]. Like our study, the Australian study used a 3D foot scanner and found men to have significantly larger values than the women for all dimensions with the exception of the first toe angle. However, men had significantly higher first and fifth toe heights and a greater fifth toe angle, and women had a significantly longer truncated foot length normalized within two common foot length categories, which is different than our results [8]. The inconsistencies between the Brazil and Australian studies and our study may be due to different measuring methods or foot categorization, or these may be true ethnic differences.

Our study results are consistent with previous studies on young or mixed-age populations. Krauss et al. showed that, for the same shoe size, young women had lower insteps than young men [1]. In addition, they found that women had smaller widths of the heel and the forefoot [1]. Aml et al. also compared foot measurements in the same foot-length category and observed that foot width and perimeter were greater in males than in females [26]. Furthermore, Wunderlich et al. normalized their data to the foot length and reported women’s feet had smaller values for the height of the first toe and the perimeter of the instep [10].

When comparing arch height between men and women, results vary between studies. The results of our study are consistent with the study by Frey [5] who reported that women presented with flatter feet than men did. Hashimoto et al. [27] who used radiography, a more reliable measurement method, to verify arch height in young adults also noted that the women had lower arches than the men. Structural changes in the female body may lead to pronation of the foot. Compared to men, women have narrower shoulders, hips are in a more varus position, and knees are in a more valgus position, which induces a pronation of the rear feet [5].

On the other hand, our results are different from 2 other studies: a survey of 441 individuals 1–80 years of age by Staheli et al. [28] that used the arch index and indicated that males have flatter feet, and a study by Zifchock et al. [29] of 145 individuals 18–65 years of age that reported that standing AHI was not significantly different but ARI was significantly different between men and women. These inconsistencies may be related to ethnic, cultural, measurement tool and age differences.

It is acknowledged that, even though subjects were instructed to distribute their body weight equally when standing so that the assessed foot supported 50 %, we could not control this with accuracy. Therefore, there may be variations in percentage of weight bearing, and as a result, different standing NH and AHI in standing position. Tessem et al. previously reported that the amount of asymmetry in weight distribution between extremities during relaxed standing is 4 % or less in healthy subjects [30]. Moreover, the sample used in this study was possibly more active or mobile due to excluding people reliant on walking sticks during the measurement.

## Conclusion

Overall, the current study provided evidence of anthropometric foot variations of older men and women. The dissimilarities are primarily in instep height, instep girth, ball girth and navicular height. Shoe manufacturers should consider the gender differences in feet when designing shoes for older adults to accommodate the greater ball and instep girth and the instep and navicular height of men’s feet and the greater first toe angle in women. Since the P value for standing heel width is also near 0.001, we recommend shoe designers also consider this difference. It remains to be seen, however, whether a shoe designed for the elderly based on gender differences suggested here would be perceived subjectively as being a better fit and, therefore, more comfortable. Further research should investigate how footwear designed according to gender differences affects fall risk.

## Abbreviation

NH: Navicular height
ND: Navicular drop
AHI: Arch height index
ARI: Arch rigidity index
